# Automatic Classification of Anomalous ECG Heartbeats from Samples Acquired by Compressed Sensing

**DOI:** 10.3390/bioengineering11090883

**Published:** 2024-08-31

**Authors:** Enrico Picariello, Francesco Picariello, Ioan Tudosa, Sreeraman Rajan, Luca De Vito

**Affiliations:** 1 Department of Engineering, University of Sannio, 82100 Benevento, Italy; epicariello@unisannio.it (E.P.); fpicariello@unisannio.it (F.P.); itudosa@unisannio.it (I.T.); 2 Department of Systems and Computer Engineering, Carleton University, Ottawa, ON K1S 5B6, Canada; sreeramanr@sce.carleton.ca

**Keywords:** wearable health device (WHD), machine learning, Internet of Medical Things (IoMT), ECG, ensemble classifier, compressed ECG classification

## Abstract

In this paper, a method for the classification of anomalous heartbeats from compressed ECG signals is proposed. The method operating on signals acquired by compressed sensing is based on a feature extraction stage consisting of the evaluation of the Discrete Cosine Transform (DCT) coefficients of the compressed signal and a classification stage performed by means of a set of k-nearest neighbor ensemble classifiers. The method was preliminarily tested on five classes of anomalous heartbeats, and it achieved a classification accuracy of 99.40%.

## 1. Introduction

According to the World Health Organization (WHO), 17.9 million people die each year due to cardiovascular disease (CVDs), and four out of five of these deaths are due to heart attacks and strokes. In contrast, one-third of these deaths occur prematurely in people under 70 years of age. Identifying those at the highest risk of CVDs and ensuring they receive appropriate treatment can prevent premature deaths [1]. Typically, to diagnose a CVD or cardiac abnormality, an electrocardiogram (ECG) signal is acquired in a clinical setting, then manually analyzed by an experienced cardiologist. However, some CVDs, such as arrhythmia, may not appear in a short trace of ECG and may require extended monitoring. Holter monitors are used for extended monitoring as subjects do their normal activities. However, such monitors are bulky and inconvenient to use, may restrict the activities that can be performed, and require hospital visits when electrodes detach from the skin. With the advent of miniaturized wearable health devices (WHDs), continuous monitoring of ECG signals is possible. WHDs can be implemented on small-footprint circuits with low power consumption in a fabric, and the wireless sensor network can be integrated into a garment [2], making them comfortable and not bulky and a convenient alternative to Holter monitors [3,4,5]. However, as WHDs are implemented using low-power microcontrollers with limited memory and battery power, only a limited amount of data can be acquired and stored. This may be highly restrictive for continuous monitoring; therefore, WHDs may have to communicate to a central node for storage and manipulation of the acquired data. Since data transmission represents the most demanding task in terms of energy consumption for a WHD node [6], to deal with these limitations, the state of the art proposes the use of compression techniques to reduce the data rate toward the central node. Compression techniques or algorithms are usually divided into the following two main categories: (i) lossless and (ii) lossy algorithms. Lossless techniques, such as the Lempel–Ziv–Welch [7] and Huffman [8] methods, can compress the ECG signal without substantial loss of information but generally have Compression Ratio (CR) values of 2 or 4 (the number of samples is reduced by a factor of 2 or 4, respectively). On the other hand, lossy techniques can reach a much greater compression ratio (between 10 and 20) but with the possibility of losing portions of significant information. Among the lossy techniques for ECG compression, transformation techniques such as Discrete Wavelet Transform (DWT) [9,10,11] and Fourier transform [12] are widely used, since they can compress the signal without loss of clinical information. These techniques impose a heavy computational load on the node of the WHD, so they are not suitable for use with a wearable device. Among the lossy methods, Compressed Sensing (CS) has great potential to be used in WHDs, as it has a low computational load in the signal compression phase. Consequently, compression can be performed directly on the WHD nodes, while the reconstruction of the signal, which has a significantly higher computational requirement, can be performed by a receiver node. The main challenge in developing CS frameworks for the compression and reconstruction of ECG signals is finding compression (sensing matrix) and reconstruction (dictionary matrix) matrices that offer the best trade-off between the quality of reconstruction and achievable compression [13,14,15,16]. Besides acquiring and compressing the ECG signal, WHD can also be considered as part of a system that can detect cardiac anomalies. As anomaly detection and classification are performed in real time, a cardiologist can be promptly warned through an alert system in case of a positive detection [17].

**Figure 1:**
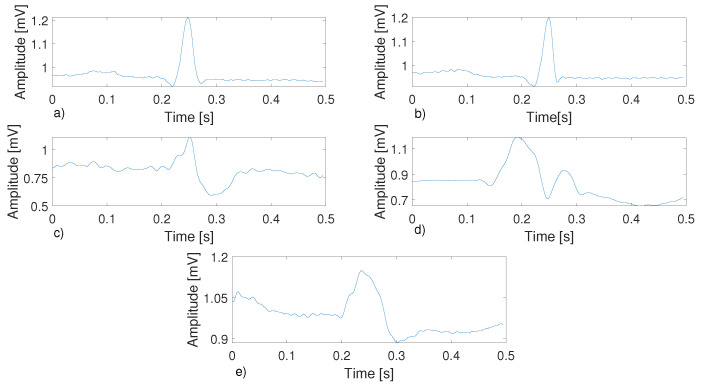
An overview of the five analyzed beats. (**a**) Normal beat; (**b**) atrial premature beat; (**c**) Right-branch block beat; (**d**) premature ventricular contraction; (**e**) left-branch block beat.

In this paper, a new method for the classification of ECG signal arrhythmias from their compressed representations obtained using CS is presented. The method works on compressed heartbeat waveforms obtained by segmenting the ECG signal according to the R-peak position and compressing it with a Deterministic Binary Block Diagonal (DBBD) matrix. The method evaluates the discrete cosine transform (DCT) coefficients of each compressed heartbeat and identifies the heartbeat type among the considered categories. For the classification of the beats, a combination of ensembled k-Nearest Neighbor (KNN) classifiers was used, working in parallel. Before validating the method in a real-life scenario with the ECG signals acquired from a wearable device, a preliminary evaluation phase was performed using signals from the MIT-BIH database to compare the performance of the proposed method with literature results. Among different datasets, the MIT-BIH database was chosen because it has been used most studies regarding ECG anomaly detection. In this preliminary phase, five heartbeat classes were considered, as they are the most commonly considered in the literature regarding ECG anomaly detection, namely Atrial Premature Beat (APB, labeled as A), Premature Ventricular Contraction (PVC, labeled as V), normal beats (labeled as N), Right-Branch Block Beat (RBBB, labeled as R), and Left-Branch Block Beat (LBBB, labeled as L), whose characteristic waveforms are shown in Figure 1.

It is important to clarify a fundamental point. This article aims to carry out the classification of ECG beats, not of the entire trace, based on tags inserted by cardiologists. This research is not intended to be a replacement for medical diagnosis but can be used as a support for diagnosis alongside the expertise of cardiologists. The following are the contributions of this paper:

- A classification methodology is proposed for distinguishing different arrhythmias in ECG signals acquired by compressed sensing using an ensemble of classifiers and a small set of features.
- The proposed methodology can perform classification without the need to reconstruct the signal. This makes it particularly suitable for use in combination with WHDs that can work with limited energy resources.

The rest of the paper is organized as follows. Section 2 reports the state of the art for ECG classification using machine learning (ML), while Section 3 provides an overview of CS. Section 4 presents the proposed method for the classification of ECG arrhythmias, while Section 5 presents the experimental evaluation and the limitations of the proposed method. Finally, Section 6 provides the conclusion and possible directions for future work.

## 2. Background

To classify the beats of the ECG signal, features need to be defined and extracted. Typically, the features considered in the state of the art can be mainly divided into the following four categories [18]: features in the time domain (e.g., Principal Component Analysis (PCA) [19,20,21,22], Linear Discriminant Analysis (LDA) [20], and Independent Component Analysis (ICA) [23,24]), features in the frequency domain (e.g., Discrete Fourier Transform (DFT) coefficients, DCT [25], and Power Spectral Density (PSD) [26]), statistical measures or measurement based on the signal morphology [27], and nonlinear methods (e.g., wavelet transform coefficients [21,22], Higher-Order Statistics (HOS) [28,29], and empirical mode decomposition [30]).

In [27], Chazal et al. used a set of time-domain features combined with the morphological features of an ECG signal. Some of the used features included pre-RR intervals, post-RR intervals, mean RR, local RR, QRS duration, T-wave duration, P-wave presence, QRS, normalized QRS, T wave, and normalized T wave. The classifier was trained on the signals from the MIT-BIH arrhythmia database, from which 44 ECG signals were extracted from different subjects without a pacemaker. The classifier model is based on linear decomposition, and 12 different classifier configurations were used. In particular, eight configurations present a classifier trained on a single ECG lead, while the other four present two classifiers trained on two ECG leads. The final classification of a beat is carried out by combining the outputs of the two classifiers with a maximum likelihood combiner. The classifier groups the ECG signals into the following five different classes defined by the Advancement of Medical Instrumentation (AAMI) guidelines: normal beats and bundle branch block beats (tagged as N), supraventricular ectopic beats (SVEBs) (tagged as S), ventricular ectopic beats (VEBs) (tagged as V), beats that result from fusing normal beats and VEBs (tagged as F), and unknown beats (tagged as Q). Considering the exhibited performance, the best achieved accuracy was 96.4%.

In [26], Plawiak used PSD obtained using the Welsh method combined with the discrete Fourier transform of the ECG signal, which was normalized by applying a logarithmic function to the transformed signal. To reduce the data, speed up the classification, and eliminate features that contain non-significant information, Rutkowski et. al. [31] proposed a system for the automatic classification of ECG signal anomalies with a feature selection phase using a Genetic Algorithm (GA). The proposed method can classify 17 types of beats, using 1000 10-second-long ECG segments from the MIT-BIH database as training signals. The method was tested using dour classifiers, namely Support Vector Machine (SVM), k-Nearest Neighbor (KNN), Probabilistic Neural Network (PNN), and Radial Basis Function Neural Network (RBFNN), managing to achieve an accuracy of 98.85%.

In [25], Roshat et al. proposed a method for the classification of ECG arrhythmias that uses the DCT features of the signal. Signals from the MIT-BIH database were used for classification. Initially, there is a signal preprocessing phase, in which a wavelet-based denoising technique is used to clean up the signal of noise. Subsequently, a Pan–Tompkins algorithm [32] is used to detect the R peak. Following this, 200 samples are taken around the peak (100 before the peak and 99 after the peak), and the DCT is applied. A PCA-based feature selection phase is used to eliminate redundant or non-significant features. The system is able to recognize five types of beats (same classes as [27]), and the method has been tested using the following six classifiers: a neural network (NN), PNN, SVM, and radial basis functions (linear, quadratic, and polynomial). The authors reported a maximum accuracy of 99.52% using a PNN classifier.

Turker et al. [33] used morphological wavelet transform features projected onto a lower dimensional feature space using PCA and temporal ECG features. The proposed classifier is a fully connected artificial neural network optimized patient-wise using a multidimensional particle swarm optimization technique. Following the AAMI guidelines, it discriminated five classes and was able to achieve an average accuracy of 98.58%.

Osowski et al. [34] proposed a neural network classifier based on hybrid fuzzy logic, using HOS as the feature set to discriminate the following seven ECG classes: normal beats, LBBB, RBBB, APB, PVC, ventricular flutter waves, and ventricular escape beats. The average accuracy is 96.06%.

Isin et. al. [35], developed a deep learning framework that was able to carry out the automatic classification and diagnosis of arrhythmias in ECG signals. The deep learning framework was trained on a general image dataset, after which it carries out automatic ECG arrhythmia diagnostics. A transferred deep convolutional neural network (namely, AlexNet) is used as a feature extractor, and the extracted features are fed into a simple back-propagation neural network to carry out the final classification. The authors used the MIT-BIH arrhythmia database as their dataset and selected three different conditions of ECG, namely (i) normal beats, (ii) paced beats, and (iii) right-branch block beats. The authors obtained a test accuracy of 92.44%.

In addition to methods and algorithmic improvements in ECG analysis, there is still room for improvement in terms of its applications, for example, in IoMT and telehealth devices [18]. The methods presented above are focused on increasing the accuracy of classification without considering that in IoMT and telemedicine applications, the devices used to acquire the signal are constrained in terms of power consumption, as they are battery-powered. This implies that in scenarios such as those described above, wearable devices cannot be used for long-term acquisition of ECG signals. Another limitation that can occur in these systems is that when multiple patients are analyzed, the transmission systems cannot support the necessary data rate. It is possible to apply compression techniques to the ECG signals and perform automatic classification directly from the compressed samples without carrying out reconstruction because the compressed signal contains all the information necessary to identify arrhythmias. This results in a reduction in power consumption compared to classical classification methods in which compression is followed by reconstruction before classification, enabling the use of wearable technologies, as, since the transmitted data are compressed, the data rate will be lower.

In [36], Alvarado et al. proposed a new method for the classification of anomalies from ECG signals for WHD applications. The signal is first preprocessed through a bank of filters to eliminate baseline wandering, and the temporal features of the signal are extracted from a compressed version of the signal. Compression is applied using a model based on the Integrate and Fire (IF) sampler. The authors analyzed the ECG signals using the stream of pulses generated by the IF sampler, extracted the pulse features, and evaluated the classifier’s performance. Therefore, the ECG signal does not need to be reconstructed. The chosen classifier is based on an LDA model, and the proposed method achieves the dual purpose of compressing the data-intensive ECG signals and performing classification in the pulse domain, following the AAMI guidelines for classification, achieving an accuracy of 93.6%.

Zheng et al. [37] proposed a method utilizing singular value decomposition (SVD) to compress ECG signals and feed the compressed data to a convolutional neural network (CNN) and SVM for classification. The system can discriminate among the following four classes: normal beats, PVC, RBBB, and LBBB. A total of 11 records were obtained from the MIT-BIH cardiac arrhythmia database and used for the training and testing phases, and a Pan–Tompkin algorithm was used to divide the ECG signals into frames (one for each heartbeat). The highest average accuracies were 99.39% with the CNN classifier and 99.21% with SVD compression.

In [38], Huang et al. developed an accurate method for the classification arrhythmias in ECG signalsthat involves compressing the signal using the maximal overlap wavelet packed decomposition in order to decompose the ECG into sub-signals of different scales. They used the Fast Compression Residual Convolutional Neural Network (FCResNet) and were able to discriminate among the following five different beat types: LBBB, RBBB, PVC, normal beats, and APB, achieving an overall accuracy of 98.79%. When comparing the performance among these three methods, it is possible to observe that the methods presented in [37,38] achieve higher accuracies than the method reported in [36], but since they are based on NNs, they have the following disadvantages: (i) the requirement of large quantities of data for training; (ii) computational complexity, which results in the use of specialized hardware; and (iii) the lack of interpretability.

## 3. Compressed Sensing

Compressed sensing is a framework aimed at acquiring and reconstructing signals that are sparse in a particular domain. Let $x\in R^{N\times 1}$ be the vector of *N* samples acquired at the Nyquist rate and $y\in R^{M\times 1}$ be its compressed acquisition; then, the CS compression process can be described as follows:

(1) y=Φ·x,

where M≪N, and $\Phi \in R^{M\times N}$ is the *sensing matrix*. The signal (x) must have a sparse representation in a specific domain (i.e., the signal can be represented by a few coefficients in the chosen domain) [15,39,40]. If this assumption is valid, then the signal can be reconstructed from its compressed representation using a relatively small number of samples [14]. The ECG signal can be represented with a sparse signal model [14]; therefore, it can be compressed using the CS approach.

The sparse representation of the ECG signal can be modeled as follows:

(2) x=Ψ·α,

where $\Psi \in R^{N\times P}$ is the dictionary matrix and $\alpha \in R^{P\times 1}$ is the coefficient vector of the signal (*x*) in the transform domain, with *P* being the number of waveforms in the dictionary. Substituting (2) into (1), the following expression can be obtained:

(3) y=Φ·x=Φ·Ψ·α.

## 4. Proposed Method

**Figure 2:**
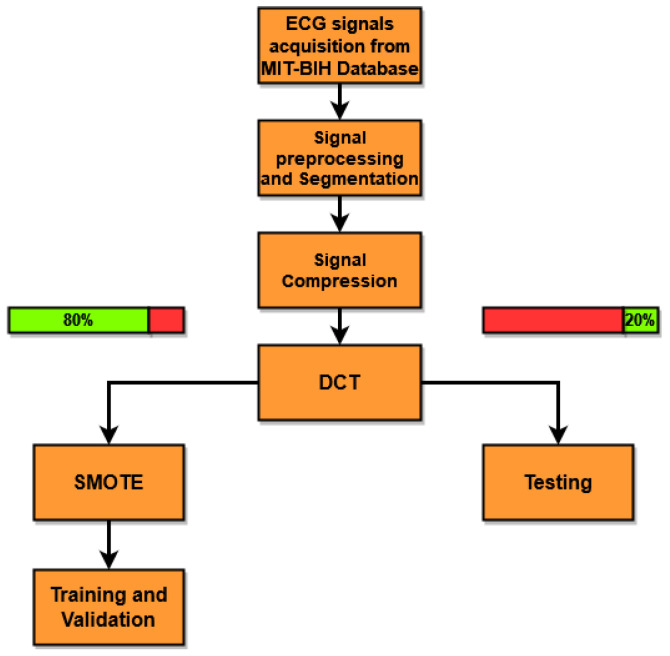
An overview of the proposed method for ECG signal acquisition, feature extraction, and unbalancing data correction.

**Figure 3:**
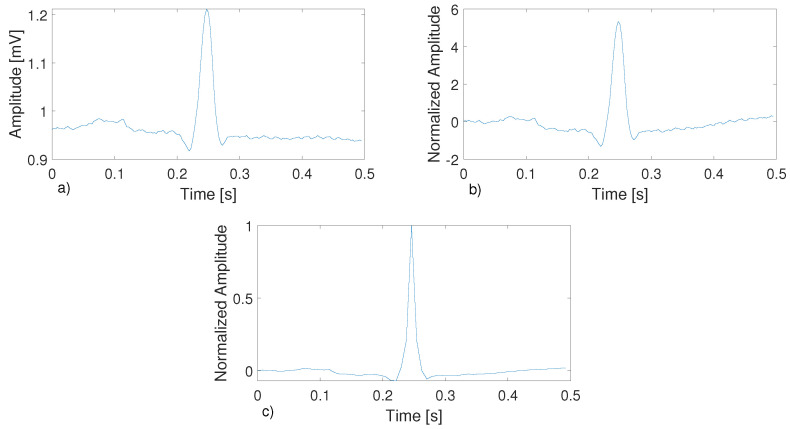
An overview of the signal segmentation, processing, and compression phases. (**a**) Segmented ECG beat; (**b**) filtered and normalized ECG beat; (**c**) compressed ECG beat.

A synthetic overview of the proposed method is presented in Figure 2 and can be summarized in the following points:

- The single-lead ECG signal is read from the database.
- The ECG signal is segmented (Figure 3a) by obtaining sub-signals containing a heartbeat and centered on the R peak and subsequently filtered (Figure 3b) to eliminate the effects of baseline drift.
- The segmented signals are compressed by employing the CS algorithm (Figure 3c).
- The DCT of the compressed ECG signals is then obtained, and all of them are used to train the classifiers.
- At this point, the dataset is divided between the training/validation and testing phases. For the training/validation phase, 80% of the signals are selected, while 20% are selected for the test phase.
- Every heartbeat is labeled by a cardiologist, and the following five different beat types are formed: normal beats (N), APB (A), PVC (V), LBBB (L), and RBBB (R). Since the majority of the classes present in the dataset are N, the Synthetic Minority Over-sampling Technique (SMOTE) algorithm is used to generate synthetic samples for the other four minority classes [41].
- The classifiers are trained with the DCT features of the compressed signals using the dataset designated for use in the training/validation phase, and in the test phase, the outputs of the trained classifiers are combined to make a decision.

### 4.1. ECG Signal Processing

The digital signal processing stage of the proposed method is based on that reported in [42]. This step can be divided into the following two phases: signal segmentation and signal filtering. In the first phase, the method uses the Pan–Tompkins algorithm [32] to identify the QRS complex of the ECG signal. As a result, the positions of the R peaks of the signal are obtained. For segmentation, windows with lengths equal to 180 samples are used, centered on the positions of the R peaks identified by the Pan–Tompkins algorithm. This involves splitting the original ECG signal into segments, each centered on the R peak and containing the QRS complex (i.e., the signals are broken down by heartbeats, and each sub-signal contains a beat). The objective of the second phase is to remove the baseline from each ECG segment. This task is accomplished through the use of two cascaded median filters; the first median filter is applied over 200 ms of ECG, and the second filter is applied over 600 ms. The obtained filtered signal is then compressed.

### 4.2. ECG Signal Compression

Following the digital signal processing stage, each ECG segment was compressed using CS as per (1). It has been already demonstrated that the ECG signal is sparse, and CS can be applied to it successfully [43]. Moreover, CS compression depends on the choice of the sensing matrix. In particular, in the case of ECG signals, it has been shown that the choice of a DBBD matrix as the sensing matrix outperforms traditional methods that use a randomly generated sensing matrix [44]. The DBBD matrix used in this work is shown in (4).

(4) $$\Phi =\left[\begin{array}{cccc} \left[1...1\right] & 0 & 0 & 0\\ 0 & \left[1...1\right] & 0 & 0\\ 0 & 0 & ... & 0\\ 0 & 0 & 0 & \left[1...1\right] \end{array}\right]$$

The DBBD matrix is sparse, and each row has a number of ones equal to $CR=N_{s}/M$, where $N_{s}$ is the number of samples of the segmented signal before compression and *M* is the number of samples of the segmented signal after compression. The reconstruction problem consists of taking the *M* compressed measurements and using both the sensing matrix (Φ) and the dictionary matrix (Ψ) to reconstruct the original signal (x). Since M≪N, there are infinite solutions to (3), but under the assumption made in this case (i.e., the signal is *K*-sparse), an estimation of the coefficient (α) can be obtained as follows:

(5) $$\hat{\alpha }=\arg\min_{\alpha }{∥\alpha ∥}_{0}\;\mathrm{subject}\;\mathrm{to}:\;y=\Phi \;\cdot \;\Psi \;\cdot \;\alpha ,$$

where ${∥\;\cdot \;∥}_{0}$ represents the $\ell_{0}$-norm operator. Equation (5) represents a constrained optimization problem, where the aim is to find the α vector as the *maximally sparse* solution, subject to (3). However, since the positions of the nonzero elements in the vector are unknown, the problem has a combinatorial complexity. For this reason, Equation (5) is relaxed to an $\ell_{1}$ optimization problem [45] as (6), which, in contrast, can be solved by linear programming as follows:

(6) $$\hat{\alpha }=\arg\min_{\alpha }{∥\alpha ∥}_{1}\;\mathrm{subject}\;\mathrm{to}:\;y=\Phi \;\cdot \;\Psi \;\cdot \;\alpha .$$

By estimating the $\hat{\alpha }$ coefficients, it is possible to reconstruct the ECG signal from the compressed samples using (2). Several algorithms can be used to reconstruct the signal [46], and typically, the most commonly used reconstruction algorithm is Orthogonal Matching Pursuit (OMP). OMP is a lossy algorithm with low complexity and computational load, but its reconstruction performance is not very high, especially at CR values higher than 4. If the arrhythmia detection system can discriminate an ECG arrhythmia from its compressed version, completely skipping the computationally expensive reconstruction of the signal for the detection of the arrhythmia, the reconstruction phase can be performed after arrhythmia detection by a receiver node instead of the WHD. In this work, the DCT is applied to the segmented and compressed ECG signal, and its coefficients are used as features by the classifiers for arrhythmia detection.

### 4.3. Balancing the Dataset

In the ML algorithm, data imbalance is an issue, as the performance of the classifier is affected by the majority class [47]. Therefore, the data need to be balanced in the training set before the classifiers are trained. This can be accomplished as part of the pre-processing phase. It can be accomplished either at the sample level or at the feature level. The most popular approach is to over-sample the training dataset, adding artificial samples to the data space; this method is called SMOTE. In SMOTE, synthetic samples are produced from the minority classes [41]. These samples are generated in the feature space by taking the minority classes and introducing synthetic samples along the line segments joining any/all of the k minority-class nearest neighbors in the feature space. Depending on the amount of over-sampling required, neighbors from the k nearest neighbors are randomly chosen. For instance, if the needed amount of over-sampling is 200%, only two among the five nearest neighbors are chosen, and one sample is generated in the direction of each. Synthetic samples are generated in the following way:

- Take the difference between the feature vector (sample) under consideration and its nearest neighbor.
- Multiply the difference by a random number between 0 and 1.
- Add the result to the feature vector under consideration. This approach forces the decision region of the minority class to be more general.

### 4.4. Training and Validation

All classifiers used in this work are members of the ensemble subspace KNN class. The application of the random subspace ensemble technique to KNN classifiers was first introduced in [48]. Being an ensemble technique, it combines the decisions of a series of weak classifiers to discriminate among various classes. The technique is derived from stochastic discrimination, where a series of stochastically created weak classifiers are combined to increase classification accuracy. Individual classifiers cannot discriminate individual classes by themselves; it is the combination of these classifiers that creates the decision-making power. In particular, the classifiers are made independent through a stochastic process; some dimensions of the feature space are ignored so that the invariance of classification is maintained for samples that differ only in the ignored dimensions. Combinations of ignored dimensions are chosen randomly so that independence is maintained. This method provides an estimate of the posterior probability (P(C|x)) of a vector (x) belonging to a class (C), and the kernels are defined by single weak classifiers—in this case, by the locations of the K nearest neighbors of a sample. This implies that the nearest neighbors can be different for each sample.

**Figure 4:**
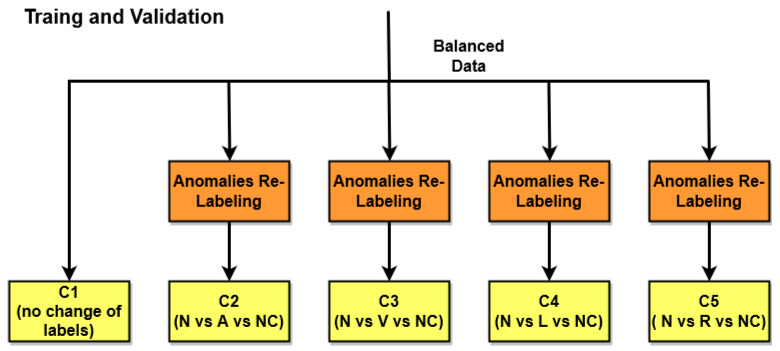
An overview of the training and validation phase.

In Figure 4, an overview of the training and validation phase is presented. For the classification, five KNN ensemble classifiers are trained using the ECG data, with a five-fold cross-validation to allow the method to discriminate among the following five different beats: normal beats (N), APB (A), PVC (V), LBBB (L), and RBBB (R). In particular, referring to Figure 4, C1 is the generic classifier, which is trained on all five types of heartbeats without making any changes to the labels. Classifiers C2, C3, C4, and C5 are also trained on the same signals as the generic classifier, but the labels are modified in such a way as to obtain 3 possible classes instead of 5. All classifiers were trained with normal beats (labeled as N) and on a different single arrhythmia for each of the classifiers (e.g., classifier C2 was trained on arrhythmia A, classifier C3 on arrhythmia V, etc.). The three remaining arrhythmias (e.g., L, R, and V in the case of C2; A, L, and R in the case of C3; etc.) were inserted into a single class labeled as NC.

### 4.5. Testing

**Figure 5:**
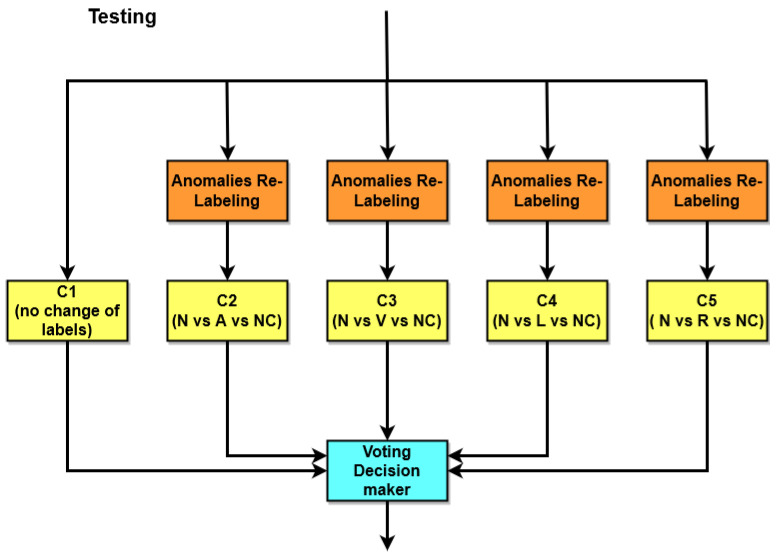
An overview of the testing phase.

The testing phase is presented in Figure 5. In this phase, the output of the individual classifiers is combined through a voting decision maker. As previously stated in Section 4.4, the possible outcomes of the four expert classifiers are normal beat (N), arrhythmia (A, V, R, or L), and unclassified arrhythmia (NC). The network implements a set of voting-based rules that allows for the choice of the most likely class. For the recognition of normal beats (class N), since each classifier can have class N as an output, a simple decision-making rule based on the majority is implemented. In the ideal case, when a normal beat occurs, all 4 classifiers should output class N. Obviously, this does not always happen; consequently, if at least 3 of the 4 expert classifiers are in agreement with class N, then the beat is classified as N (majority voting). In the case of an abnormal beat, the ideal case would be that three classifiers output NC while one classifier outputs arrhythmia, that is, all 4 classifiers recognize the presence of an arrhythmia; three cannot recognize it, and the fourth gives the arrhythmia output. This would be the same if there were three outputs of arrhythmia (two of them as NC) and one output of normal beat, in which case majority vote is still valid. In the event that a majority is not reached, the generic classifier output is taken as the default decision. In this case, the outputs of the expert classifiers are discarded.

## 5. Experimental Evaluation

To carry out the experimental evaluation of the proposed method (see Figure 2), the ECG signals were taken from the freely available online Physionet MIT-BIH Arrhythmia Database [49]. The Physionet Database contains 48 ECG tracks from 47 ambulatory patients acquired through two channels for a period of half an hour. These acquisitions were made at a sampling frequency of 360 Hz, with a resolution of 11 bits per channel. Each ECG beat present in the database has been annotated by a cardiologist. This annotation is used to discriminate among the various beats and to select the desired heartbeat based on the classes discriminated by the classifier. For these tests, 44 out of the 48 available ECG tracks were used, where the 4 left-out tracks correspond to paced beats. As described in Section 4, the QRS complexes were identified, the ECG signals were segmented into sub-signals centered on the R peak, each containing a beat of finite length equal to $N_{s}=180$ samples. Two cascaded median filters were then applied to the segmented signals to eliminate the baseline. Since each beat has a label, only the beats that fall into one of the following five distinct classes are selected: normal beats (N), APB (A), LBBB (L), RBBB (R), and PVC (V). The signals are then compressed through CS, using a DBBD matrix (4) with a compression ratio of $CR=N_{s}/M$, where *M* is the number of samples of the compressed signal. The experimental tests presented in this work were carried out by varying the CR to evaluate the classification performance and robustness of performance under various CRs. DCT is applied to the segmented and compressed signals, and its coefficients are used as features for classifier training. For the training phase, 80% of the signals for each class was selected randomly, and the remaining 20% was used for the testing phase. The SMOTE algorithm was used to handle data imbalance.

In Table 1, the numbers of signals used for (i) training and validation without SMOTE, (ii) training and validation with SMOTE, and (iii) testing are presented. As can be seen, when dealing with the MIT-BIH dataset, there is a prevalence of normal beats (N) above all others. To reduce the unbalance, SMOTE was set to double the number of samples of the other classes, namely R, L, and V. Since the samples of class A are the smallest among all classes, the multiplicative coefficient was set to 2.5 (rounded down). Since the data used for the test must not be contaminated by artificial samples, in order to make the test data remain independent from those used in the training, SMOTE is applied only to the latter. To evaluate the performance of the proposed method, five commonly used figures of merit are calculated as follows:

- Accuracy: The number of the correctly classified instances divided by the number of total instances (7);
- Sensitivity: the number of positive instances that are correctly classified divided by the sum of the number of positive instances that are correctly classified plus the number of positive instances that are wrongly classified (8);
- Specificity: the number of negative instances correctly classified divided by the sum of the number of negative instances correctly classified and the number of positive instances wrongly classified (9);
- Precision: the number of positive instances correctly classified divided by the total number of positive instances (10);
- F1 score: the harmonic mean of precision and sensitivity (11).

(7) $$Accuracy=\frac{TP+TN}{TP+TN+FN+FP}$$

(8) $$Sensitivity=\frac{TP}{TP+FN}$$

(9) $$Specificity=\frac{TN}{TN+FP}$$

(10) $$Precision=\frac{TP}{TP+FP}$$

(11) $$F1-\mathrm{score}=2\times \frac{Precision\times Sensitivity}{Precision+Sensitivity}$$

### 5.1. Training and Validation Results

In order to not disrupt the flow of the main text of the article, all the tables referred to from now on can be found in Appendix A. In Table A1, the class-wise results from the training and validation of classifier C1 are reported for different values of CR. As can be seen from the presented results, the C1 classifier performs well during validation. Furthermore, the sensitivity, specificity, precision, and F1 score are higher in almost all cases (≥99%). As expected, as the CR increases, the number of cases of misclassification increases, and this leads to a degradation of the performance of the classifier. A decrease in accuracy with the CR is expected, as the DBBD matrix behaves as a low-pass filter on the signal, and by increasing the CR, the cut-off frequency of such a filter decreases. As a consequence, the information content of the signal is reduced. However, all figures of merit, once again, remain, for most cases, above 99%.

In Table A2, the accuracy, average sensitivity, average precision, average specificity, and average F1 score are reported for C1. The performance of the classifier decreases only marginally as the CR changes. The classifier has an accuracy of 99.69% with a CR equal to 3, down to 99.60% with a CR equal to 9. Validation was carried out in the same way for the other four classifiers, and the results are presented in Table A3, Table A4, Table A5, Table A6, Table A7, Table A8, Table A9 and Table A10. In particular, Table A3 and Table A4 refer to classifier C2, Table A5 and Table A6 refer to classifier C3, Table A7 and Table A8 refer to classifier C4, and Table A9 and Table A10 refer to classifier C5. The performance is similar across all classifiers.

### 5.2. Testing Results

The results of the test phase are shown in Table A11, Table A12 and Table A13. In Table A11, the performance of the C1 classifier is reported, with similar results to what was presented in the validation phase. The figures of merit are calculated for each class and at different values of CR, while in Table A12, the average performance of the classifier is shown. Again, as the CR changes, the combination of compressed DCT features and the ensemble classifier is robust, as the performance degradation due to the higher compression is only marginal. Classes A and V appear to be those with the most classification errors, and they are also the most affected by the increase in CR. In fact, class A attains a sensitivity of 90.18% with CR = 3, and this degrades to 87.92% with CR = 9, while for class V, the sensitivity degradation spans from 98.26% with CR = 3 to 96.30% with CR = 9. In particular, performance degradation is evident for class A. Referring to Figure 1a,b, it is possible to note that the differences between a beat labeled as N and one labeled as A are minimal. This leads to very similar DCT coefficients, and consequently, it is harder for the classifier to discriminate between the two beat types. Classes N, R, and L have results similar to those seen in validation, with sensitivity, precision, and F1 scores higher than 99% for every CR value. Analyzing the average values in Table A12, the accuracy of the classifier remains above 99% for all CR values, presenting a maximum of 99.39% with CR = 3. Table A13 shows the results of the classifier after the decision maker. It is observed that for a CR equal to 3, the decision maker is unable to improve the performance of the classifier. However, as the CR value increases, it is possible to notice an increase in accuracy. In particular, in comparing the values between Table A12 and Table A13, the same performance as the classifier without the decision maker is achieved by the classifier with the decision maker using the next CR value utilized in the test. Following the use of the decision maker, the maximum accuracy of the proposed method is 99.40%, while the minimum is 99.15%. In Table A14, a performance overview of studies regarding the classification of ECG signals is presented. All these studies use the MIT-BIH arrhythmia dataset, and the data size is also reported. Since in [26,38], the exact number of ECG beats was not reported, because both of the methods work with ECG 10 s segments chosen randomly from the database, an estimation of the ECG beats is reported, considering one heartbeat per second. In particular, in [33,37,38], the classification of the ECG signal was carried out on its compressed representation. It can be noted that the proposed method has an accuracy in line with that reported in [25], which is one of the highest values reported in the literature, while sensitivity was 1.27% lower, precision was 1.10% lower, and specificity was 0.23% lower. The proposed method outperforms all other classification methods using compressed ECG signals and is comparable with the method presented in [38] in terms of accuracy. However, it should be noted that in [38], class A was not used. Carrying out the same previously presented tests with only the N, L R, and V classes, the accuracy of the proposed method was determined to be 99.74%.

### 5.3. Limitations of the Proposed Method

Although the performance of the method in terms of accuracy is comparable with that of methods that do not use ECG signal compression and with those that use neural networks, it presents limitations listed in this subsection and that will be the subject of subsequent studies and future works. The method was tested for the classification of five types of beats, and not all possible arrhythmias or variations of arrhythmias were present in the dataset. The choice to use these five classes was made because they are the most used classes in the literature, with the aim of being able to validate the method and compare it with previously developed methods. The tests were carried out using signals that were acquired in medical scenarios, but these signals were not acquired by wearable devices. This means that in a scenario where a wearable is used, the presented accuracies could decrease, for example, due to motion artifacts. In this case, a possible solution is to carry out a quality assessment phase on the ECG signal to discard beats that are too corrupted by movement artifacts. Even though the method does not feature the use of NNs, it still uses five classifiers. This implies that the employed dataset must be large enough to ensure correct training of the method. Since the number of DCT features used to train the classifiers depends on the CR used to compress the ECG signals, they decrease as the CR increases. This implies a decrease in the duration of the training phase, but this means that every time a user wants to change the CR of the method, the classifiers must be retrained.

## 6. Conclusions

In this paper, a method for classifying compressed ECG beats is presented to be used in a WHD-based telemedicine system. The method operates based on heartbeats compressed by compressed sensing using a DBBD sensing matrix. To evaluate the performance of the classifier, accuracy, sensitivity, specificity, precision, and F1 score were calculated and compared with those of other classification methods found in the literature. In validation, the proposed method obtained an accuracy of 99.72%, a sensitivity of 99.74%, a precision of 99.71%, a specificity of 99.84%, and an F1 score of 99.73% for a CR value of 3, while these values dropped to 99.62%, 99.63%, 99.65%, 99.76%, and 99.64%, respectively, for a CR value of 9. In testing, an accuracy of 99.40%, a sensitivity of 97.42%, a precision of 98.48%, a specificity of 99.68%, and an F1 score of 97.96% for a CR value of 3 were observed, while for CR value of 9, these values dropped to 99.15%, 96.57%, 98.14%, 99.53%, and 97.15%, respectively. From these results, it is possible to conclude that the use of the KNN ensemble classifier and the DCT coefficients of the compressed signal is a robust solution for the classification of ECG signals in the compressed domain, without the need to reconstruct the signal. Comparison of the obtained results with the state of the art shows that the proposed method achieves comparable performance to that of methods that do not use compression and out-performs other methods classifying compressed ECG signals while only using the compressed DCT coefficients of the ECG signal. Future work will aim to improve the performance of the classifier, as it is clear from the tests that most of the classification errors are due to classes A and V. In particular, the use of other features that can be obtained from the compressed signal, such as PSD, should be taken into consideration. In addition, the decision maker will be improved to make a reasonable contribution, even with a low value of the CR. Furthermore, the system will be tested in a real-life scenario with the use of ECG signals acquired through the ATTICUS WHD reported in [50] in both medical and real-life activity scenarios.

## Figures and Tables

**Table 1:** Number of signals used for (i) training and validation before the SMOTE algorithm, (ii) training and validation after the SMOTE algorithm, and (iii) testing.

N° of Signals					
Classes	N	A	R	L	V
**Before SMOTE**	59,622	2037	5806	6458	5522
Total	79,415				
**After SMOTE**	59,622	7130	11,612	12,916	11,044
Total	102,324				
**Test**	14,905	509	1451	1615	1380
Total	19,860				

## Data Availability

Data used in this study are publicly available at https://www.physionet.org/content/mitdb/1.0.0/ accessed on 4 June 2022.

## Abbreviations

The following abbreviations are used in this manuscript:

ECG	ElectroCardioGram
DCT	Discrete Cosine Transform
WHO	World Health Organization
CVD	CardioVascular Disease
CR	Compression Ratio
CS	Compressed Sensing
DBBD	Deterministic Binary Block Diagonal
APB	Atrial Premature Beat
PVC	Premature Ventricular Contraction
RBBB	Right-Branch Block Beat
LBBB	Left-Branch Block Beat
KNN	k-Nearest Neighbor
NC	Not Classified
ML	Machine Learning
PCA	Principal Component Analysis
LDA	Linear Discriminant Analysis
ICA	Independent Component Analysis
DFT	Discrete Fourier Transform
PSD	Power Spectral Density
HOS	Higher-Order Statistics
AAMI	Advancement of Medical Instrumentation
SVEB	SuperVentricular Entropic Beat
VEB	Ventricular Entropic Beat
GA	Genetic Algorithm
SVM	Support Vector Machine
PNN	Probabilistic Neural Network
NN	Neural Network
FCResNet	Fast Compression Residual convolutional neural Network
OMP	Orthogonal Matching Pursuit

## Appendix A. Tables

### Appendix A.1. Validation Tables

**Table A1:** Validation results for classifier C1 against different values of CR divided by class.

Normal Beat (Class N)				
CR	Sensitivity	Precision	Specificity	F1
3	0.9971	0.9982	0.9975	0.9976
5	0.9970	0.9984	0.9978	0.9977
**7**	0.9968	0.9976	0.9966	0.9972
9	0.9971	0.9969	0.9957	0.9970
**Atrial Premature Beat (Class A)**				
3	0.9905	0.9834	0.9987	0.9869
5	0.9914	0.9830	0.9987	0.9872
7	0.9878	0.9822	0.9987	0.9850
9	0.9851	0.9855	0.9989	0.9853
**Left-Branch Block Beat (Class L)**				
3	0.9990	0.9991	0.9999	0.9990
5	0.9991	0.9991	0.9999	0.9991
7	0.9986	0.9988	0.9998	0.9987
9	0.9981	0.9981	0.9997	0.9981
**Right-Branch Block Beat (Class R)**				
3	0.9984	0.9991	0.9999	0.9987
5	0.9983	0.9987	0.9998	0.9985
7	0.9980	0.9991	0.9999	0.9985
9	0.9976	0.9988	0.9998	0.9982
**Premature Ventricular Contraction (Class V)**				
3	0.9961	0.9937	0.9992	0.9949
5	0.9961	0.9934	0.9992	0.9947
7	0.9939	0.9921	0.9990	0.9930
9	0.9926	0.9922	0.9991	0.9924

**Table A2:** Average sensitivity, average precision, average specificity, average F1 score, and accuracy obtained in validation for classifier C1.

CR	Accuracy	Sensitivity	Precision	Specificity	F1
3	0.9969	0.9962	0.9947	0.9990	0.9954
5	0.9969	0.9964	0.9945	0.9991	0.9954
**7**	0.9962	0.9950	0.9940	0.9988	0.9945
9	0.9960	0.9941	0.9943	0.9986	0.9942

**Table A3:** Validation results for classifier C2 against different values of CR divided by class.

Normal Beat (Class N)				
**7**	**Sensitivity**	**Precision**	**Specificity**	**F1**
3	0.9969	0.9984	0.9977	0.9976
5	0.9968	0.9984	0.9978	0.9976
**7**	0.9965	0.9974	0.9964	0.9969
9	0.9965	0.9972	0.9961	0.9968
**APC (Class A)**				
3	0.9910	0.9811	0.9987	0.9860
5	0.9912	0.9837	0.9988	0.9874
7	0.9863	0.9829	0.9987	0.9846
9	0.9850	0.9924	0.9978	0.9850
**Remaining Arrhythmias (Class NC)**				
3	0.9984	9.9977	0.9988	0.9939
5	0.9984	0.9973	0.9986	0.9978
7	0.9977	0.9968	0.9983	0.9972
9	0.9974	0.9962	0.9980	0.9968

**Table A4:** Average sensitivity, average precision, average specificity, average F1 score, and accuracy obtained in validation for classifier C2.

7	Accuracy	Sensitivity	Precision	Specificity	F1
3	0.9970	0.9957	0.9924	0.9984	0.9939
5	0.9970	0.9955	0.9931	0.9984	0.9943
**7**	0.9962	0.9935	0.9924	0.9978	0.9929
9	0.9960	0.9930	0.9924	0.9977	0.9929

**Table A5:** Validation results for classifier C3 against different values of CR divided by class.

Normal Beat (Class N)				
**7**	**Sensitivity**	**Precision**	**Specificity**	**F1**
3	0.9970	0.9983	0.9977	0.9976
5	0.9973	0.9984	0.9977	0.9978
**7**	0.9967	0.9972	0.9961	0.9969
9	0.9971	0.9970	0.9958	0.9970
**PVC (Class V)**				
3	0.9962	0.9929	0.9991	0.9945
5	0.9961	0.9929	0.9992	0.9945
7	0.9931	0.9911	0.9989	0.9921
9	0.9914	0.9918	0.9990	0.9916
**Remaining Arrhythmias (Class NC)**				
3	0.9973	9.9960	0.9982	0.9966
5	0.9974	0.9965	0.9984	0.9969
7	0.9963	0.9960	0.9982	0.9961
9	0.9957	0.9958	0.9981	0.9957

**Table A6:** Average sensitivity, average precision, average specificity, average F1 score, and accuracy obtained in validation for classifier C3.

7	Accuracy	Sensitivity	Precision	Specificity	F1
3	0.9970	0.9968	0.9957	0.9983	0.9969
5	0.9972	0.9969	0.9959	0.9984	0.9964
**7**	0.9962	0.9954	0.9948	0.9977	0.9950
9	0.9961	0.9947	0.9949	0.9976	0.9948

**Table A7:** Validation results for classifier C4 against different values of CR divided by class.

Normal Beat (Class N)				
**CR**	**Sensitivity**	**Precision**	**Specificity**	**F1**
3	0.9970	0.9984	0.9978	0.9977
5	0.9986	0.9981	0.9974	0.9983
**7**	0.9967	0.9975	0.9965	0.9971
9	0.9971	0.9971	0.9959	0.9971
**LBBB (Class L)**				
3	0.9990	0.9994	0.9999	0.9992
5	0.9990	0.9988	0.9998	0.9989
7	0.9984	0.9985	0.9998	0.9984
9	0.9981	0.9981	0.9997	0.9981
**Remaining Arrhythmias (Class NC)**				
3	0.9970	9.9939	0.9975	0.9954
5	0.9963	0.9945	0.9977	0.9954
7	0.9951	0.9934	0.9973	0.9942
9	0.9942	0.9943	0.9977	0.9942

**Table A8:** Average sensitivity, average precision, average specificity, average F1 score, and accuracy obtained in validation for classifier C4.

7	Accuracy	Sensitivity	Precision	Specificity	F1
3	0.9972	0.9977	0.9972	0.9983	0.9974
5	0.9971	0.9980	0.9971	0.9983	0.9975
**7**	0.9964	0.9967	0.9965	0.9974	0.9966
9	0.9964	0.9965	0.9965	0.9978	0.9965

**Table A9:** Validation results for classifier C5 against different values of CR divided by class.

Normal Beat (Class N)				
**7**	**Sensitivity**	**Precision**	**Specificity**	**F1**
3	0.9971	0.9985	0.9979	0.9978
5	0.9970	0.9983	0.9977	0.9976
**7**	0.9967	0.9973	0.9962	0.9970
9	0.9967	0.9971	0.9959	0.9969
**RBBB (Class R)**				
3	0.9981	0.9990	0.9999	0.9985
5	0.9984	0.9989	0.9999	0.9986
7	0.9985	0.9991	0.9999	0.9988
9	0.9977	0.9990	0.9999	0.9983
**Remaining Arrhythmias (Class NC)**				
3	0.9970	9.9940	0.9974	0.9955
5	0.9966	0.9938	0.9973	0.9954
7	0.9948	0.9936	0.9972	0.9942
9	0.9946	0.9934	0.9971	0.9940

**Table A10:** Average sensitivity, average precision, average specificity, average F1 score, and accuracy obtained in validation for classifier C5.

7	Accuracy	Sensitivity	Precision	Specificity	F1
3	0.9972	0.9974	0.9971	0.9984	0.9973
5	0.9970	0.9973	0.9970	0.9983	0.9972
**7**	0.9964	0.9967	0.9967	0.9978	0.9967
9	0.9962	0.9963	0.9965	0.9976	0.9964

### Appendix A.2. Testing Tables

**Table A11:** Test results for classifier C1 against different values of CR divided by class.

Normal Beat (Class N)				
**7**	Sensitivity	Precision	Specificity	F1
3	0.9981	0.9954	0.9861	0.9967
5	0.9973	0.9948	0.9843	0.9960
**7**	0.9974	0.9940	0.9820	0.9957
9	0.9976	0.9932	0.9794	0.9954
**APB (Class A)**				
3	0.9018	0.9464	0.9987	0.9245
5	0.8900	0.9283	0.9982	0.9087
7	0.8861	0.9357	0.9984	0.9102
9	0.8782	0.9371	0.9984	0.9067
**LBBB (Class L)**				
3	0.9963	0.9969	0.9997	0.9966
5	0.9963	0.9926	0.9993	0.9944
7	0.9957	0.9938	0.9995	0.9947
9	0.9913	0.9913	0.9992	0.9913
**RBBB (Class R)**				
3	0.9917	0.9972	0.9998	0.9944
5	0.9924	0.9959	0.9997	0.9941
7	0.9917	0.9945	0.9996	0.9931
9	0.9931	0.9959	0.9997	0.9945
**PVC (Class V)**				
3	0.9826	0.9869	0.9990	0.9847
5	0.9754	0.9883	0.9991	0.9818
7	0.9710	0.9857	0.9990	0.9743
9	0.9630	0.9844	0.9989	0.9736

**Table A12:** Average sensitivity, average precision, average specificity, average F1 score, and accuracy obtained in the testing phase for classifier C1.

CR	Accuracy	Sensitivity	Precision	Specificity	F1
3	0.9939	0.9741	0.9846	0.9967	0.9794
5	0.9926	0.9703	0.9800	0.9961	0.9750
**7**	0.9921	0.9684	0.9807	0.9957	0.9743
9	0.9908	0.9646	0.9804	0.9951	0.9723

**Table A13:** Average sensitivity, average precision, average specificity, average F1 score, and accuracy obtained in the testing phase after the voting decision maker.

CR	Accuracy	Sensitivity	Precision	Specificity	F1
3	0.9940	0.9742	0.9848	0.9968	0.9796
5	0.9934	0.9719	0.9832	0.9964	0.9774
**7**	0.9924	0.9727	0.9825	0.9958	0.9760
9	0.9915	0.9657	0.9814	0.9953	0.9715

**Table A14:** Comparison of the proposed method with other state-of-the art ECG classification methods.

ECG Classification without Compression						
**Study**	**Accuracy**		**Sensitivity**	**Precision**	**Specificity**	**Data Size (Beats)**
P. de Chazal et. al. [27]	96.4		77.5	90.6	N/A	109,492
Plawiak [26]	98.75		89.35	N/A	99.39	about 10,000 (estimated)
R.J. Martis et. al [25]	99.52		98.69	99.58	99.91	110,094
**ECG Classification with Compression**						
**Study**	**CR**	**Accuracy**	**Sensitivity**	**Precision**	**Specificity**	**Data Size**
Turker I. et. al. [33]	20	98.30	84.60	87.40	98.70	83,648
Huang et. al. [38]	1.85	98.79	95.16	99.39	N/A	About 25,000 (estimated)
Zheng et. al. [37]	2	99.39	N/A	N/A	N/A	20,000
**Proposed**	**3**	**99.40**	**97.42**	**98.48**	**99.68**	**122,184**
**Proposed (Classes N, L, R, and V)**	3	99.74	99.46	99.47	99.83	114,545
